# Low Prevalence of *Pneumocystis* pneumonia (PCP) but High Prevalence of *Pneumocystis* dihydropteroate synthase (*dhps*) Gene Mutations in HIV-Infected Persons in Uganda

**DOI:** 10.1371/journal.pone.0049991

**Published:** 2012-11-16

**Authors:** Steve M. Taylor, Steven R. Meshnick, William Worodria, Alfred Andama, Adithya Cattamanchi, J. Lucian Davis, Samuel D. Yoo, Patrick Byanyima, Sylvia Kaswabuli, Carol D. Goodman, Laurence Huang, International HIV-associated Opportunistic Pneumonias (IHOP) Study

**Affiliations:** 1 Department of Epidemiology, Gillings School of Global Public Health, University of North Carolina, Chapel Hill, North Carolina, United States of America; 2 Division of Infectious Diseases and International Health, Duke University Medical Center, Durham, North Carolina, United States of America; 3 Makerere University-University of California, San Francisco Research Collaboration, Kampala, Uganda; 4 Department of Medicine, Makerere College of Health Sciences, Mulago Hospital, Kampala, Uganda; 5 Division of Pulmonary and Critical Care Medicine, San Francisco General Hospital, University of California San Francisco, San Francisco, California, United States of America; 6 Health Tutors’ College Mulago, Kampala, Uganda; 7 Department of Laboratory Medicine, San Francisco General Hospital, San Francisco, California, United States of America; 8 HIV/AIDS Division, San Francisco General Hospital, University of California San Francisco, San Francisco, California, United States of America; Instituto de Higiene e Medicina Tropical, Portugal

## Abstract

*Pneumocystis jirovecii* pneumonia (PCP) is an important opportunistic infection in patients infected with HIV, but its burden is incompletely characterized in those areas of sub-Saharan Africa where HIV is prevalent. We explored the prevalence of both PCP in HIV-infected adults admitted with pneumonia to a tertiary-care hospital in Uganda and of putative *P. jirovecii* drug resistance by mutations in fungal dihydropteroate synthase (*dhps*) and dihydrofolate reductase (*dhfr*). In 129 consecutive patients with sputum smears negative for mycobacteria, 5 (3.9%) were diagnosed with PCP by microscopic examination of Giemsa-stained bronchoalveolar lavage fluid. Concordance was 100% between Giemsa stain and PCR (*dhps* and *dhfr*). PCP was more prevalent in patients newly-diagnosed with HIV (11.4%) than in patients with known HIV (1.1%; p = 0.007). Mortality at 2 months after discharge was 29% overall: 28% among PCP-negative patients, and 60% (3 of 5) among PCP-positive patients. In these 5 fungal isolates and an additional 8 from consecutive cases of PCP, all strains harbored mutant *dhps* haplotypes; all 13 isolates harbored the P57S mutation in *dhps*, and 3 (23%) also harbored the T55A mutation. No non-synonymous *dhfr* mutations were detected. PCP is an important cause of pneumonia in patients newly-diagnosed with HIV in Uganda, is associated with high mortality, and putative molecular evidence of drug resistance is prevalent. Given the reliability of field diagnosis in our cohort, future studies in sub-Saharan Africa can investigate the clinical impact of these genotypes.

## Introduction

*Pneumocystis jirovecii* pneumonia (PCP) is a major opportunistic infection in people with HIV and AIDS. In the United States and Europe, its incidence has decreased dramatically among HIV-infected patients owing to the twin interventions of antimicrobial prophylaxis of high-risk patients and combination antiretroviral (ARV) therapy. However, in sub-Saharan Africa, access to these interventions remains limited for the 23 million people living with HIV infection; recent estimates indicate that the prevalence of PCP amongst HIV-infected patients with pneumonia may be as high as 27% in some African countries [1].

PCP prophylaxis decreases the risks of PCP, PCP-related death, and all-cause death amongst patients with AIDS. [2] However, putative *P. jirovecii* drug resistance threatens the durability of PCP prophylaxis with antifolate antibiotics, which are the mainstay of PCP prevention amongst high-risk patients. Antifolate drugs target two enzymes that are necessary for the fungus to synthesize folate – dihydropteroate synthase (DHPS) and dihydrofolate reductase (DHFR) – and mutations in the genes encoding these proteins (*dhps* and *dhfr*, respectively) are associated with reduced *in vitro* drug efficacy. [3], [4] Because drug efficacy cannot be directly assessed in the absence of a reliable *P. jirovecii* cultivation system, and because the presence of several mutations is consistently associated with exposure to antifolates, [5] these mutations may represent a genetic signature of clinical drug resistance. In the United States and Europe, the prevalence of two of the most common *dhps* mutations (those at codons 55 and 57) in patients with PCP has been reported between 23–81% (in the USA) and 7–57% (in Europe). [6] Estimates of the independent effect of *P. jirovecii* mutants upon clinical outcomes have been conflicting: some studies report their association with increased mortality or antifolate-based treatment failure, [7]–[10] while others have not [11]–[13].

Despite the potential high burden of PCP in sub-Saharan Africa, few studies have explored *P. jirovecii dhps* and *dhfr* genotypes in HIV-infected patients in this region, and no studies have done so outside of Southern Africa. [14], [15] We investigated *P. jirovecii* among a cohort of HIV-infected patients admitted to Mulago Hospital, a national referral center in Kampala, Uganda. We assessed the prevalence both of PCP amongst patients with pneumonia and of *P. jirovecii* mutations associated with antifolate drug resistance. We hypothesized that PCP would be common and that mutations would be associated with reported exposure to antifolates.

## Methods

### Ethics Statement

The study was approved by institutional review boards at Mulago Hospital and Makerere University, the University of California, San Francisco, the University of North Carolina at Chapel Hill, and by the Uganda National Council for Science and Technology. All patients provided written informed consent.

### Patient Enrollment and Sample Collection

For this study, *P. jirovecii* molecular testing was employed in two phases in an effort to first assess the prevalence of PCP amongst HIV-infected patients with pneumonia and subsequently to assess the prevalence of mutant *P. jirovecii* genotypes among patients with microscopically-confirmed PCP while also optimizing resources. In phase 1, we tested consecutively-enrolled patients meeting the criteria below between September 2007 and July 2008, to determine the prevalence of PCP at Mulago Hospital and to allow for comparison of *Pneumocystis* detection by microscopy and PCR. After we found a low prevalence of PCP in phase 1, in phase 2, we obtained molecular sequence data from only those patients with microscopically-diagnosed PCP between September 2008 and October 2009.

Details of the clinical study have been described previously. [16] Briefly, consecutive patients that were admitted to Mulago Hospital were screened; patients were offered enrollment if they were HIV-positive, had cough ≥2 weeks but <6 months, and received a clinical diagnosis of pneumonia. All patients had a chest x-ray and provided two sputum samples for examination for acid-fast bacilli (AFB) by direct Ziehl-Neelsen microscopy. Clinical outcome was assessed either in person or by telephone interview at 2 months after discharge.

Patients with sputa negative for AFB were referred for bronchoscopy. Two experienced pulmonologists performed bronchoscopies by completely visualizing central airways and lavaging the radiographically-most-affected lobe of the lung with up to 125 mL of sterile normal saline. Trained laboratory technicians in the Microbiology Department at Mulago Hospital performed all initial examinations of BAL fluid, including modified Giemsa stain for *P. jirovecii*; Giemsa-stained BAL fluid was subsequently assessed by a second reader at San Francisco General Hospital who was masked to other clinical and laboratory data. Aliquots of 1–2 mL of unprocessed BAL fluid were frozen at −20°C for subsequent molecular analyses.

### Molecular Methods

PCR testing was performed at the University of North Carolina by personnel masked to clinical and microscopic diagnoses. BAL specimens were thawed and briefly vortexed. Genomic DNA (gDNA) was extracted from 200 µL of each BAL specimen using the EZ1 Virus Mini Kit v2.0 (Qiagen, Valencia, CA). Individual samples were amplified in nested PCR assays targeting the *dhps* and *dhfr* genes of *P. jirovecii*. [17], [18] Products were electrophoresed on 1% agarose gels, with amplification confirmed by the presence of a single band at 330 base pairs (for *dhps*) or at 850 base pairs (for *dhfr*). Separate work areas for gDNA extraction, reaction set-up, and electrophoresis were maintained to minimize contamination of the PCR testing, and all reaction plates included negative and positive controls with either molecular-grade water or gDNA from a patient with known PCP, respectively.

Samples demonstrating amplification were purified with the QIAquick Gel Extraction Kit (Qiagen). Purified products were bidirectionally sequenced using ABI BigDye Terminator chemistry at the UNC Core Sequencing Facility. Sequences were aligned with Sequencher v4.8 (Gene Codes, Ann Arbor, MI). Wildtype *P. jirovecii* sequences obtained from GenBank were used as referents for alignments: U66282 for *dhps* and AF090368 for *dhfr*. Base calls at loci corresponding to codons 55 and 57 of *dhps* were confirmed by manual inspection of the chromatograms of contigs that were built from both forward and reverse sequencing reads. Aligned sequences were manually inspected both for mixed alleles (defined as peaks ≥10% of primary peak, above baseline) and for mutations at other loci (including those in the sequenced fragment of *dhfr*); novel mutations were defined as single nucleotide polymorphisms evident in both sequences obtained from the same amplicon at a locus not described above.

### Definitions and Statistical Analyses

Haplotypes for *dhps* were assigned based upon codons 55 and 57: “Wildtype” refers to isolates with wildtype alleles at both loci, “single-mutant” to isolates with a wildtype allele at only one locus, and “double-mutant” to isolates with mutant alleles at both loci. For haplotype assignment, mixed alleles were classified as mutant.

We compared demographic and clinical data between patients with and without PCP with the student’s t-test or Kruskal-Wallis test for continuous variables, or with the chi-squared test for categorical variables. We compared agreement between PCP diagnosis by modified Giemsa stain and PCR using Cohen’s kappa. All statistical analyses were conducted with Stata/IC (version 10, Stata Corp., College Station, TX).

## Results

### Prevalence of PCP

In phase 1 overall, 407 consecutive patients were enrolled and 218 (54%) were eligible for bronchoscopy; 132 patients (32%) ultimately underwent bronchoscopy and BAL. Therefore, 129 patients were investigated in phase 1 (three specimens were missing), and clinical characteristics were similar between those who did and did not undergo bronchoscopy. [19] Mean (SD) age of these 129 patients was 34 years (9.1), and 73 (57%) were women. Ninety-four patients (73%) had been previously diagnosed with HIV. Among these 94 patients, 22 (23.4%) were taking antiretrovirals on admission, and 77 (81%) reported taking PCP prophylaxis (76 with trimethoprim-sulfamethoxazole, 1 with dapsone). The median (interquartile range) CD4 cell count among 128 patients with available values was 74 cells/mm^3^ (17–192), and 98 patients (77%) had a CD4 cell count below 200 cells/mm^3^.

As previously described, the most frequent pulmonary diagnoses in these patients who underwent bronchoscopy were pulmonary tuberculosis (TB), pulmonary cryptococcosis, pulmonary Kaposi sarcoma, and PCP. [20] By microscopic evaluation of modified Giemsa-stained BAL specimens, five patients (3.9%) were diagnosed with PCP. Two patients were diagnosed with PCP alone, and one patient each was also co-infected with pulmonary *Cryptococcus neoformans*, culture-positive pulmonary tuberculosis, or culture-negative pulmonary tuberculosis. Four of the five were newly diagnosed with HIV; all five had CD4 cell counts below 50 cells/mm^3^, and none were taking antiretrovirals or PCP prophylaxis. Overall mortality at 2 months after discharge was 29% (38 of 129): 28% of non-PCP patients had died (35 of 124), compared with 60% of PCP patients (3 of 5; Risk Ratio 2.13; 95% C.I. 0.98–4.59).

Five patients (3.9%) were positive in the both of the PCR assays targeting *dhps* and *dhfr*. Agreement between detection by microscopy and PCR was 100% (p<0.001). Additionally, agreement between the reader at Mulago Hospital and the second, masked reader at San Francisco General Hospital was also 100%.

### Prevalence of dhps and dhfr Genotypes

In total, 13 *dhps* and *dhfr* sequences were available (5 from phase 1 and an additional 8 from phase 2) (Table 1). All 13 isolates harbored the P57S mutation in *dhps*; in one specimen, the mutation was present in addition to the wildtype allele. Only 3 (23%) harbored the T55A mutation; in one specimen, the mutation was present with the wildtype allele.

**Table 1:** Prevalence of *P. jirovecii* genotypes.

	No. (%) of patients (n = 13)
***dhps*** ** mutant codons**	
T55A	3 (23.1)
P57S	13 (100)
***dhps*** ** haplotypes**	
Wildtype	0
Single mutant	10 (76.9)
Double mutant	3 (23.1)
***dhfr*** ** haplotypes**	
Wildtype	12 (92.3)
Mutant*	1 (7.7)

*dhps*: dihydropteroate synthase; *dhfr*: dihydrofolate reductase. Patients with mixed alleles were classified as mutant.

* The single *dhfr* polymorphism encoded a synonymous mutation at base pair 1437 of the reference sequence AF090368.

The single-mutant *dhps* haplotype was present in 10/13 (77%) specimens, and the double-mutant *dhps* haplotype was present in the remaining three. Thus, there were no wildtype *dhps* haplotypes present.

In 12 of 13 specimens *dhfr* was wildtype across the sequenced loci. One specimen demonstrated a synonymous thymidine to cytosine substitution at base pair 1437 of the reference sequence. Thus, none of the isolates harbored mutant DHFR enzymes.

### Clinical and Molecular Correlates

There were no significant differences in the mean age or gender distribution of patients with and without PCP (Table 2). All patients with PCP had a CD4 count <200 cells/mm3; median CD4 cell count was lower among patients diagnosed with PCP (12 cells/mm3) than without (88; p = 0.033 by Kruskal-Wallis). No PCP patients were taking antiretrovirals on admission.

**Table 2:** Comparison of 129 consecutive HIV-infected patients with and without Pneumocystis pneumonia (PCP).

	PCP-positive (n = 5)	PCP-negative (n = 124)	p-value*
**Age**, yr, mean (SD)	35.2 (8.9)	34.0 (9.1)	0.78
**Women**, no. (%)	2 (40)	71 (57.3)	0.45
**CD4 cell count**, cells/µl, median (interquartile range)	12 (10–39)	88 (22–196)	0.033
**CD4 cells<200** cells/µl, %	5 (100)	93 (75.6)	0.21
**New HIV diagnosis**, no. (%)	4 (80)	31 (25)	0.007
**Taking PCP prophylaxis** **, no. (%)	0 (0)	77 (82.8)	0.03
**Receiving antiretrovirals** **, no. (%)	0 (0)	22 (23.7)	0.58
**Positive PCR assay**, no. (%)			
*dhps*	5 (100)	0	NA
* dhfr*	5 (100)	0	NA

HIV: human immunodeficiency virus; PCR: polymerase chain reaction; *dhps*: dihydropteroate synthase; *dhfr*: dihydrofolate reductase; NA: not applicable.

* Determined by the student’s t-test or Kruskal-Wallis test for continuous variables, or the chi-squared test for categorical variables.

** Among 94 patients with previously-diagnosed HIV.

PCP was significantly more prevalent among the 35 patients newly diagnosed with HIV (11.4%) than the 94 patients with previously-diagnosed HIV (1.1%; p = 0.007). Among the 94 patients with known HIV, PCP was diagnosed in 1 of 17 (5.9%) not taking PCP prophylaxis and 0 of 77 who reported receiving PCP prophylaxis (p = 0.032).

Among the 13 total patients with PCP, the *dhps* double mutant was present in 1 of 2 patients taking PCP prophylaxis (both with trimethoprim-sulfamethoxazole) and 2 of 11 (18.2%) not taking prophylaxis (p = 0.326). Only 1 *dhfr* mutant was identified, this in a patient not taking PCP prophylaxis. The absence of wildtype *dhps* genotypes precluded a meaningful assessment of the effect of *dhps* genotype upon clinical PCP outcome.

## Discussion

In this cross-sectional study of HIV-infected Ugandans admitted to a tertiary care hospital with pneumonia who underwent bronchoscopy, the prevalence of *Pneumocystis* pneumonia was low, but all *P. jirovecii* isolates harbored mutations in the *dhps* gene. These mutations are putatively associated with antifolate resistance based upon epidemiologic evidence and experience with other pathogens. Additionally, the field diagnosis of PCP using Giemsa staining demonstrated excellent concordance with molecular diagnosis. Because PCP remains an important opportunistic infection in HIV-infected patients that are not receiving PCP prophylaxis, these findings have important implications for the care of patients with AIDS in sub-Saharan Africa.

Our data indicate that *P. jirovecii* is now an uncommon etiology of pneumonia in hospitalized, HIV-infected patients in Kampala, Uganda. Reported prevalences of PCP in sub-Saharan African settings vary widely, including 4% in the Central African Republic, [21] 5% in Bujumbura, [22] 5% in Kigali, [23] 8% in Dakar, [21] 8% in Moshi, [24] 27% in Blantyre, [1] 30% in Addis Ababa, [25] and 33% in Harare. [26] Indeed, the prevalence of PCP (diagnosed using an immunofluorescent antibody test) was 39% among patients admitted to Mulago Hospital and enrolled with similar criteria to ours between 1999 and 2000. [27] Importantly, only 25% of patients in the earlier cohort reported by Worodria *et al.* reported receiving cotrimoxazole prior to admission, [27] while in our cohort, 60% of all patients and 81% of those who were previously-diagnosed with HIV received PCP prophylaxis. Our study was not designed to assess the efficacy of PCP prophylaxis, but among patients with known HIV, we do note a significant negative association between PCP diagnosis and the receipt of PCP prophylaxis. Therefore, the successful provision of PCP prophylaxis to HIV-infected patients in Kampala that comprised our cohort may have reduced the overall prevalence of PCP.

Despite the low overall PCP prevalence, among patients newly-diagnosed with HIV, the prevalence of PCP was 11.4%, indicating that PCP remains a significant clinical concern in those newly-diagnosed with HIV or not on PCP prophylaxis. Our study highlights the applicability of microscopic examination of modified Giemsa-stained pulmonary specimens for field diagnosis of PCP: Agreement was 100% between this technique and PCR at the *dhps* and *dhfr* loci. Several studies in high income settings have described the performance characteristics of the modified Giemsa method in comparison with staining with Grocott’s methenamine silver and toluidine blue-O or direct and indirect immunofluorescence staining, [28], [29] but its performance has not been formally reported from low- and middle-income settings, where the burdens of AIDS and PCP are greatest. Though immunofluorescent staining is most commonly employed for diagnosis in high income settings, its expense precludes its wide use in many low- and middle-income settings. Our data suggest that the less-expensive modified Giemsa stain is a viable option for reliable PCP diagnosis in settings with high prevalences of HIV in settings such as sub-Saharan Africa.

All patients in our study harbored mutant *P. jirovecii dhps* isolates. Though this may derive from a small sample size, this is notable both because it exceeds prevalences reported in other regions and because most patients individually denied prior exposure to PCP prophylaxis with antifolate drugs, which is commonly associated with the presence of *P. jirovecii* mutations. [5] Prevalences of mutant *P. jirovecii dhps* genotypes vary geographically, [6], [30] and are generally highest in high income settings. [31] Fewer reports have reported prevalences from sub-Saharan Africa, though 56% of isolates from South Africa in 2006 harbored mutant *dhps* haplotypes. [14] The high prevalence in our study despite the lack of individual antifolate PCP prophylaxis may derive from two non-exclusive processes: interhuman transmission of mutant strains or population-level selective pressure exerted by antifolate use for other indications. Interhuman transmission of mutant strains has been inferred from ecological studies of mutant genotypes in which geographic residence was associated with genotype. [30] Though the factors that mediate the transmission of *P. jirovecii* remain incompletely understood, two additional findings in Uganda argue against this explanation. Specimens with mutant *dhps* haplotypes were not clonal, as we detected 3 different haplotypes of the *P. jirovecii* mitochondrial large subunit of ribosomal DNA in specimens with identical mutant *dhps* haplotypes. [16] Additionally, *P. jirovecii* colonization – one putative reservoir for interhuman transmission – was rare in our cohort, with only 6% of patients with non-Pneumocystis pneumonia harboring *P. jirovecii* DNA as measured by a different genomic marker that amplifies a multi-copy gene as opposed to the single-copy *dhps* and *dhfr* genes. [16] Taken together, the non-clonality of mutant strains and the low prevalence of *P. jirovecii* colonization suggest that interhuman transmission was not primarily responsible for the high prevalence of mutant *Pneumocystis*.

Notably, all patients in our study harbored *Pneumocystis* strains with the *dhps* P57S mutation, and this near-fixation of the P57S mutation suggests significant population-level selective pressure. Notably, the pattern of mutant haplotypes – with a preponderance of single-mutant P57S haplotypes – is similar to that observed in a study from France, wherein a high prevalence of these haplotypes was associated with the use of sulfadoxine-pyrimethamine (SP, or Fansidar) as prophylaxis as compared with the use of trimethoprim-sulfamethoxazole (co-trimoxazole). [32] SP use is prevalent is Uganda: until recently, SP was recommended first-line therapy for malaria, and at the time of this study was still commonly used for treatment of suspected malaria. [33] Taken together, the previous literature linking SP and the P57S mutation, the high prevalence of the P57S mutation in our study, and the widespread use of SP in Uganda during this period, suggest that sulfadoxine may differentially select for the P57S mutation in the *dhps* gene of *P. jirovecii*.

We observed no non-synonymous mutations in the sequenced segment of *dhfr*. Mutations in *P. jirovecii dhfr* vary greatly in prevalence and allele between published studies, [12], [18], [34] and in some settings non-synonymous mutations have been associated with the receipt of pyrimethamine-containing PCP prophylaxis. [35] Though we failed to detect any such mutations, the small sample size of *dhfr* haplotypes in our cohort precludes inferences of selection pressure on *P. jirovecii dhfr* haplotypes in Uganda.

Our study has several limitations. The high prevalence of PCP prophylaxis use likely reduced the prevalence of PCP in our cohort and may fetter comparison with other settings where PCP prevalence is higher. Additionally, though concordance between microscopic and molecular diagnosis was excellent, the use of BAL specimens in our study may limit the generalizability of this observation. Furthermore, a number of eligible patients did not undergo bronchoscopy owing to patient refusal, death, or the establishment of an alternate firm diagnosis, [20] and so the true prevalence of PCP cannot be determined conclusively. Clinical data that are commonly employed to gauge the likelihood of PCP – including serum lactate dehydrogenase, arterial blood gas, chest radiograph – were not uniformly obtained, precluding assessment of their relationship to microscopically-confirmed PCP in this setting. Finally, the limited number of fungal genotypes prevented a fuller characterization of the relationship between clinical and molecular data and continued study is warranted.

Our study of pneumonia amongst HIV-infected Ugandans highlights the need to consider PCP as an etiology of pneumonia in sputum AFB smear-negative patients with newly-diagnosed HIV and those not on PCP prophylaxis in Uganda, and describes a clinically- and cost-effective method for field diagnosis. Additionally, we describe the near-fixation of a specific *P. jirovecii dhps* mutation, which may be associated with patterns of sulfa antibiotic use. Given the high prevalence of this haplotype which is associated with drug resistance and the large population at risk of PCP in sub-Saharan Africa, future studies are required to more definitively characterize the clinical impact of these mutations.
